# A Consensus-Reaching Approach to the Evaluation of Product Design Alternatives with Multiple Preference Structures

**DOI:** 10.1155/2021/6992648

**Published:** 2021-11-10

**Authors:** Yukun Hu, Suihuai Yu, Jianjie Chu, Dengkai Chen, Fangmin Cheng, Chen Chen, Zhuo Liu, Ting Wei

**Affiliations:** ^1^Key Laboratory of Industrial Design and Ergonomics, Ministry of Industry and Information Technology, Northwestern Polytechnical University, Xi'an 710072, China; ^2^TUT School of Art & Design, Tianjin University of Technology, Tianjin 300384, China; ^3^College of Art and Design, Shaanxi University of Science & Technology, Xi'an 710021, China

## Abstract

With interdisciplinarity being an important characteristic of contemporary product design, the evaluation of design alternatives also involves multiple disciplines, and the evaluator group usually consists of evaluators from different fields and with obvious heterogeneous characteristics. To effectively satisfy the heterogeneous needs of evaluators and improve the credibility of evaluation results, the paper introduces a consensus-reaching approach that incorporates multiple preferences to the evaluation of product design alternatives. First, in order to obtain individual preference information, each evaluator is asked to evaluate all the design alternatives using a preference structure that he/she is familiar with. Second, we use a transfer function to uniform the evaluation information obtained from various preference structures into a complementary judgment matrix. Then, we use the Hybrid Weighted Averaging (HWA) operator weight determination model to aggregate the preference information and obtain the group preference information. Then, we measure the consensus degree between individual evaluators and the group using a consensus measurement method. After that, we use the feedback mechanism to instruct individual evaluators to modify their preferences until a consensus is achieved. We explain the application steps and the feasibility of this approach through the evaluation of the design alternatives of multichannel fluorescence immunochromatography analyzers (MFIAs).

## 1. Introduction

In today's fast-developing global market, industrial design has become a holistic, interdisciplinary, and integrated design activity. As a result, in the evaluation of product design alternatives, multiple disciplines are concerned, and the heterogeneity among evaluators is prominent due to differences in ethnic culture, social experience, knowledge background, cognition, etc. [1–5]. To capture and visualize the cognitive process of participants and maximize the cognitive consistency between individuals, the customized individual semantics (CIS) based on heterogeneous subordinate positive information (SPI) was constructed [6]. Chen et al. customized individual semantics by means of the possibility distribution of attitude and modeling the heterogeneity of evaluators which reflected the individual differences of cognitive styles [7]. Meanwhile, due to the difference in educational backgrounds [8–11], knowledge [12, 13], experiences [8, 10–14], cultures [8, 10, 13, 14], cognitive degrees [8, 11], attributes [15], motivations [15], personalities [15], and expression habits [10, 16] of evaluators, they usually use heterogeneous preference representation structures to express their preference for alternatives [8–16]. The preference structure is supposed to meet the heterogeneous needs of evaluators, yet a single preference structure fails to meet the differential expression habits and needs of evaluators from different fields.

The evaluation of product design alternatives is usually conducted by a group of engineers, marketers, users, managers, etc. Due to the differences in subjective preferences, background knowledge, and experience, reaching a consensus is difficult. However, it is desirable that all or at least most of the participators are satisfied with the best alternative [17–21]. The consensus-reaching process refers to the practice of the evaluator group in the process of discussing and coordinating their opinions during the evaluation process until they come to an agreement. The consensus process involves the measurement of consensus and the coordination of nonconsensus [22]. Such process is helpful for reducing cognitive disagreements during the evaluation process and improving the credibility of the design alternative. Many consensus-reaching approaches with multiple preference structures are proposed by researchers [10]. At present, consensus measures are usually calculated by the opinions given by experts or the choice degrees of alternatives obtained from these opinions. The situation may not reflect the information about the true consistency because the same alternative ranking may have different choice degree vectors.

These are questions that we need to answer: How do we ensure that the heterogeneity needs of evaluators are satisfied in the evaluation? How do we consider the consistency degree of evaluators' opinions? How do we make sure that the evaluator group can reach a certain degree of consensus on the pros and cons of the design alternatives and decide on a design alternative that is acceptable to the group? How do we verify the approach?

To address the above questions, we propose an approach that incorporates multiple preference structures to the evaluation of product design alternatives. The purpose of this paper is to meet the needs of evaluators having heterogeneous characteristics during the evaluation of design alternatives, to optimize the evaluation process, and to make the evaluation results more credible. The rest of the paper is organized as follows: Section 2 reviews the literature that discusses the evaluation and selection of product design alternatives and the group consensus process; Section 3 proposes and elaborates the approach to reach a consensus in the evaluation of alternatives with multiple preference structures; Section 4 presents a numerical example to explain the detailed process of the proposed method; Section 5 summarizes the paper and discusses the significance and limitations of the proposed method.

## 2. Literature Review

### 2.1. The Evaluation and Selection of Product Design Alternatives

We review the evaluation and selection of product design alternatives from four aspects, i.e., indicator system, product design alternatives generation, decision-making methods being applied, and the real-life application scenarios for product design alternatives selection.

#### 2.1.1. Indicator System

Researchers established different indicator systems according to the focus of the problems they solved. In the context of environmental sustainability, Shidpour et al. classified the most important factors in the key areas determined by life cycle assessment and obtained three qualitative criteria, that is, “safety,” “functional satisfaction,” and “aesthetic,” and three quantitative criteria, that is, “cost,” “reliability,” and “time to market.” The weight of each criterion was obtained by the fuzzy analytic hierarchy process [23]. To ensure the quality of products and meet the requirements of customers, Fan et al. determined the evaluation indicator system from the four dimensions of economy, functionality, innovation, and environment and applied the determined indicator system to yacht design evaluation [24]. From the point of product sustainability performance, Feng and Mai established a six-dimension sustainability evaluation model, which included environment (greenhouse emission, waste), economy (energy efficiency, material utilization, operation cost), society (health and wellness, operational safety), functionality (life, modularity, maintenance), manufacturability (processes technology, assembly, storage), and reusability (reuse, recycle, disposability), aiming to help designers and engineers evaluate and compare the relative sustainability performance of different products [25]. Shu and Zhong proposed evaluating the manufacturability of products from three aspects: economy, technology, and comprehensiveness to support the rapid response design task [26].

#### 2.1.2. Product Design Alternatives Generation

Kang et al. combined fuzzy theory, similarity theory, and ant colony optimization to complete the generation and evaluation of design schemes [27]. Zhang et al. proposed a design model for multidisciplinary oriented complex product system and developed a vector-based mapping tool to support the rapid mapping to support conceptual design [28]. Gopsill et al. generated an automatic and evolving design structure matrix by monitoring the changes of the digital models that represent the products, which completed the identification and monitoring of product components, as well as supporting the change generation of existing product life cycle solutions [29]. Based on the Naïve Bayes cluster and rough set theory, Li et al. proposed a new product concept generation method driven by customer requirements, which helped the product development team obtain customer requirements and product attributes [30]. Hsiao et al. extracted the appearance characteristics of products based on the morphological analysis method and generated the alternative set of product appearance design through the combination of appearance components for subsequent evaluation [31]. Zamani et al. proposed a hybrid schedule generation scheme to the resource-constrained project scheduling problem, called the Polarized Adaptive Scheduling Scheme, which can operate in the spectrum between two poles, that is, to generate schemes through parallel and serial schedule [32]. In addition, there were innovative scheme generation methods based on the theory of innovative problem solving (TRIZ) [33], product design generation methods based on case-based decision theory [34], etc.

#### 2.1.3. Decision-Making Methods

Guo and Ji took the metal handles of doors and windows as an example. The author used the analytic hierarchy process to calculate the weight of each goal and attribute from all design alternatives and obtained the pros and cons of each alternative through comprehensive calculation, thereby assisting decision-makers in making decisions [35]. Based on Analytical Network Process (ANP) and the modified technology for order preference by similarity to ideal solution (TOPSIS), Ayağ proposed a concept evaluation method in new product development. ANP method is used to determine the relative weight of a set of quantitative and qualitative evaluation criteria, and the improved ideal solution is used to sort the conceptual schemes according to the evaluation criteria to obtain the best scheme that meets customer expectations and company requirements [36]. Zhu et al. proposed an analytic hierarchy process that was based on the rough number to determine the weight of each evaluation criterion. They then used an improved comparison ranking method based on rough numbers to evaluate conceptual product designs. They verified the robustness of the method through the concept selection of a lithography tool [37]. Yumoto et al. proposed a decision support system for product selection based on the analytic hierarchy process, which used the decision rules of rough sets for qualitative evaluation. They verified the method through examples of used cars and sneakers [38]. Besharati et al. proposed a comprehensive design concept evaluation method based on fuzzy-technique for order preference by similarity to an ideal solution. A new “weighting criteria” was developed for the investigation process to quantify the evaluation criteria. It helps engineers to improve the effectiveness and objectivity of sustainable product development [39]. In addition, to improve the credibility of evaluation results, more hybrid methods have been developed [40]. To obtain an optimal design alternative, Huang et al. proposed several methods for the evaluation of conceptual designs under different conditions, which were based on computational intelligence, such as physical planning, genetic algorithms, neural networks, and fuzzy logic [41]. Tian et al. proposed a framework for the evaluation of design alternatives based on the analytic hierarchy process, gray correlation method, and ideal solution similarity ranking method and verified the effectiveness of the framework by applying it to the green performance evaluation of refrigerators [42].

#### 2.1.4. The Real-Life Application Scenarios for Product Design Alternatives Selection

Rossi and Sihn described a comprehensive method of life cycle-oriented product alternatives evaluation and decision support, which promoted the identification effectiveness of product concepts that meet existing constraints within a given time period, and selected the most appropriate product scheme that meets the requirements set [43]. Josh and Gupta proposed an Advanced-Remanufacturing-To-Order-Disassembly-To-Order system to evaluate the design scheme of end-of-life (EOL) and the impact of product design on recycling [44]. Park and Seo discussed the approximate life cycle assessment of the product design scheme represented by solid models in the collaborative design environment and then developed a knowledge-based approximate life cycle assessment system to assess the environmental impact of product design schemes [45].

Since product design alternatives often involve multidisciplinary knowledge, when using the mathematical calculation method, the cognitive differences among evaluators from different knowledge backgrounds need to be considered. Only when a high degree of consensus is reached can the credibility and adaptability of the final design alternative be ensured. At present, there is still a lack of research on the measurement of consensus degree and its coordination method in the process of product design evaluation. In previous studies, researchers tended to consider the same form of preference information being presented by evaluators while neglecting the influence the heterogeneity of evaluators has on preference expression. Since there are differences in the evaluators' knowledge structure, judgment level, and personal preferences, the same form of preference representation cannot address the heterogeneous needs of the evaluators.

### 2.2. Consensus-Reaching Process in Group Evaluation

The consensus-reaching process is an effective decision-making tool to eliminate preference conflicts in group decision-making [46]. The consensus-reaching process refers to the process of evaluators discussing and coordinating their differing views, reaching a certain sense of agreement prior to the decision-making [47]. It mainly involves two steps: the measurement of group consensus and the correction of nonconsensus. Since the measurement of consensus directly determines the progress of the group decision-making procedure, it is always a hot issue to calculate group consensus effectively. It is found from existing researches that the calculation of group consensus usually includes two steps: the measurement and the aggregation of preference similarity [48]. Recently, in view of the characteristics of actual decision-making problems, some measurement methods have been proposed. For example, Meng et al. introduced a consensus indicator based on Manhattan Distance Measure to measure the agreement degrees of the decision-makers' opinions in group decisions and applied it to the model selection of Enterprise Resource Planning (ERP) software [49]. Zhang et al. measured the individual consensus levels and the group consensus levels by calculating the distance between each individual interval fuzzy preference relations (IFPRs) and the collective IFPR. A feedback mechanism considering experts' leadership and the bounded confidence levels of experts was proposed to guide experts to modify their opinions [50]. Zhao et al. used the distance between individual preferences to calculate the degree of similarity and proposed a feedback mechanism based on the degree of similarity in order to find inappropriate preferences and provide guidance to the modification and eventually reach a consensus [51]. Zhang et al. measured individual consensus degree and group consensus degree by calculating the distance between individual decision matrices and collective decision matrices and developed two optimization models. They also generated adjustment advice for decision-makers who must change their opinions in the process of reaching consensus and demonstrated the use of consensus-reaching algorithm through an example of ERP system supplier selection [52]. Zhang et al. proposed some distance measurement methods between intuitionistic multiplicative numbers/sets, including improved Hamming distance and improved Euclidean distance and its weighted forms, and further developed a new intuitionistic multiplicative preference relationship consensus measure to assist the decision-making process. The author then verified the proposed method through the selection of investment projects [53]. Mata et al. defined the similarity function based on the information center of the fuzzy set and pointed out the defect of using the traditional Euclidean distance to define the similarity function [54]. To measure the preference similarity level, Chen et al. proposed a similarity measure between intervals of linguistic two tuples and a weighted average method of interval linguistic two tuples [55]. According to existing research, the ordered weighted averaging (OWA) operator is commonly used to aggregate preference similarity [56, 57]. For example, Herrera et al. introduced a consensus and consistency-based induced ordered weighted averaging operator to aggregate the preferences of evaluators in the consensus-reaching process when the fuzzy preference relations are incomplete and then applied this operator to the consensus model as well as the selection process [58]. Palomares et al. extended the OWA aggregation operators and proposed the Attitude-OWA operator, the consensus model that incorporates the group's attitudes toward consensus into the consensus measurement. The authors then applied the method to the process of solving a decision problem with different attitude definitions [59]. Peláez et al. proposed an elective aggregated majority ordered weighted averaging operator (SAM-OWA); the SAM-OWA operator calculates the weight of each value on the satisfaction measurement table by counting the number of votes each value obtained [60].

However, the characteristic of the OWA operator is that the weight has nothing to do with the data. It simply associates weight with the position of the data, i.e., the order of the data. Considering the interdisciplinarity of product design, the heterogeneity of evaluators, and the complexity of evaluation indicators, it is inadequate to only consider the order of design proposals being evaluated. Thus, the paper adopts the HWA operator which not only considers the positions of each data but also considers the importance of the data itself. The operator rearranges the data in descending order and assigns a weight to each date according to the position and the importance of each data. It takes both the importance of position and the importance of data into account, with no ignorance of each individual factor. In this way, the HWA operator effectively avoids the situation where the order of the design alternatives has to be distorted when only taking into consideration the positions or importance of the alternatives. It can reduce the influence of some nonobjective factors on the aggregation results in the aggregation process, so as to make the aggregation result more robust and reasonable.

## 3. The Proposed Consensus-Reaching Approach

**Figure 1:**
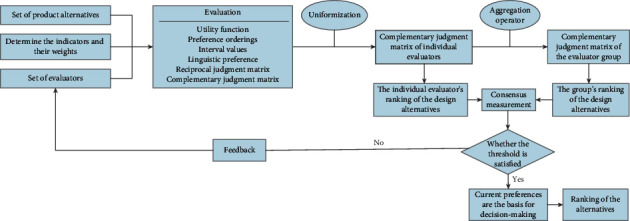
Method flow.

To meet the needs of evaluators with heterogeneous characteristics and improve the accuracy of evaluation results, the paper proposes an approach for reaching a consensus on product design alternatives with multiple preference information. The six important layers of the method (see Figure 1) are as follows: (1) determining the evaluation indicators, their weights, and the weight of the evaluator, (2) the evaluator choosing a preference representation form to express his/her initial or adjusted preferences, (3) uniforming different preference representation forms, (4) aggregating individual preferences into group preferences using aggregation operator, (5) using consensus measurement method to obtain the degree of consensus between individual evaluators and the evaluator group, and (6) using a feedback mechanism to guide evaluators to modify their preferences and then repeating steps (2) to (6) until the predefined consensus level is reached. Finally, we calculate the ranking of the alternatives.

To address the consensus-reaching problem on the evaluation of product design alternatives, we assume that the set of design alternatives to be evaluated is *X* = {*x*_1_, *x*_2_,…, *x*_*n*_}(*n* ≥ 2), the evaluator group set is *E* = {*e*_1_, *e*_2_,…, *e*_*l*_}(*l* ≥ 2), and the evaluation indicator set is *I* = {*i*_1_, *i*_2_,…, *i*_*m*_}(*m* ≥ 2), where *n*, *l*, and *m* represent the number of programs, the number of evaluators, and the number of evaluation indicators, respectively. The evaluator weight set is *W*=(*w*_1_, *w*_2_,…,*w*_*l*_)^*T*^, where *w*_*k*_ is the weight of the evaluator *e*_*k*_, *w*_*k*_ ≥ 0, *k* = 1, 2, ···, *l*, and ∑*w*_*k*_=1; the evaluation indicator weight set is *Z*=(*z*_1_, *z*_2_,…,*z*_*m*_)^*T*^, where *z*_*j*_ is the weight of the evaluation indicator *i*_*j*_, *z*_*j*_ ≥ 0, *j* = 1, 2,…, *m*, and ∑*z*_*j*_=1. At the same time, the consensus level threshold is set *γ*. When the group consensus level reaches this threshold, the evaluator group reaches a consensus on the design alternative, and the evaluation result has a high degree of reliability. Otherwise, it is necessary to identify the evaluators who have disagreements and instruct them using the feedback mechanism to modify their preferences. Based on these assumptions, the proposed approach is established with seven steps as follows.

**Step 1 .**

Determining the evaluation indicators and rating their relative importance.

For the design alternatives to be evaluated, the evaluator group discusses and establishes an evaluation indicators system and uses the analytic hierarchy process (AHP) method to calculate both the weights of individual evaluators and the weights of indicators.

AHP is an indicator-system-based decision-making method that combines qualitative and quantitative decision-making methods [61]. The weights of the evaluation indicators calculated by this method are more accurate and more consistent [62]. Thus, this paper uses the AHP method to calculate the weights of the evaluation indicators.

For the indicator set of the design alternatives to be evaluated, the weights of every two design alternatives are compared by evaluators. The values 1, 3, 5, 7, and 9 are used to indicate the importance of indicator *i* relative to indicator *j*, and the meaning of the numerals is a follows: 1 means equally important, 3 means weakly important, 5 means essentially important, 7 means relatively important, and 9 means absolutely important. The obtained judgment matrix of the indicator set is

(1) $$\begin{array}{c} A=\left[\begin{array}{ccc} a_{11} & \cdots & a_{1n}\\ \vdots & \; & \vdots \\ a_{n1} & \cdots & a_{nn} \end{array}\right]. \end{array}$$

When *i* = *j*, *a*_*ij*_ = 1; when *i* ≠ *j*, *a*_*ij*_=1/*a*_*ji*_. Then, the weight of indicator *i* is

(2) $$\begin{array}{c} w_{i}=\frac{{\left(\prod_{j=1}^{n}a_{ij}\right)}^{\left(1/n\right)}}{\sum_{i=1}^{n}{\left(\prod_{j=1}^{n}a_{ij}\right)}^{\left(1/n\right)}}. \end{array}$$

To ensure the reliability of the weight calculation, the consistency index (*CI*) and the consistency ratio (*CR*) are used for judgment, where CI=(*λ*_max_ − *n*)/(*n* − 1), CR=CI/RI, where *RI* is a random index whose value can be obtained by a table [63].*λ*_max_=∑((*A*_*c*_ · *W*^*T*^)/(*n* · *W*)) is the maximum eigenvalue of the matrix. If CI ≤ 0.1 and CR ≤ 0.1 are satisfied, then, the judgment matrix has a good consistency and the weight calculation result is valid; otherwise, it needs to be judged again.

**Step 2 .**

Evaluation of design alternatives.

To fix a set of alternatives in a design alternatives evaluation problem, there are multiple preference representation forms that can be adopted by evaluators to present their preferences for certain sets of alternatives or the information form that he/she is more familiar with. Then, we assume the experts' preferences over the set of alternatives *X* against the set of the indicators *I*, and it may be represented in one of the following six ways.

1. *Utility Function* [64]. Suppose that the evaluator *e*_*k*_ evaluates the set of alternatives *X* under a certain indicator and gives a utility value set of the alternatives, *U*^*k*^={*u*_1_^*k*^, *u*_2_^*k*^,…, *u*_*n*_^*k*^}, *u*_*i*_^*k*^ ∈ [0,1], where *u*_*i*_^*k*^ represents the utility value of the alternative *x*_*i*_ given by the evaluator *e*_*k*_. And we assume that the larger the utility value *u*_*i*_^*k*^, the better the corresponding design alternative *x*_*i*_
2. *Preference Orderings* [65]. Suppose that the evaluator *e*_*k*_ evaluates the alternative set *X* under a certain indicator and gives the order of the alternative as *O*^*k*^={*o*_1_^*k*^, *o*_2_^*k*^,…, *o*_*n*_^*k*^} in which a ranking vector from the best to the worst is obtained
3. *Interval Values* [66]. Suppose that the evaluator *e*_*k*_ evaluates the set of alternatives *X* under a certain indicator and gives the evaluation vector of alternative *x*_*i*_, vector *d*_*i*_^*k*^=[*d*_*i*_^*l*^*k*^^, *d*_*i*_^*u*^*k*^^], where *d*_*i*_^*k*^=[*d*_*i*_^*l*^*k*^^, *d*_*i*_^*u*^*k*^^] is the interval number, *d*_*i*_^*l*^*k*^^ and *d*_*i*_^*u*^*k*^^ are real numbers, and *d*_*i*_^*l*^*k*^^ < *d*_*i*_^*u*^*k*^^
4. *Linguistic Preference* [67, 68]. Suppose that the evaluation given by the evaluator *e*_*k*_ to the set of alternatives *X* under a certain indicator is described by a matrix *V*^*k*^. *v*_*ij*_^*k*^ can be understood as the degree to which alternative *x*_*i*_ is better than alternative *x*_*j*_ and the degree is an element selected from the predefined linguistic evaluation set *L*={*l*_0_, *l*_1_, *l*_2_,…, *l*_*g*/2_, *l*_(*g*/2)+1_,…, *l*_*g*_}, where there are *g*+1 elements in the set *L.* Its corresponding subscript *i* can be obtained by function *I*, *I* : *L*⟶*N*, *I*(*l*_*i*_)=*i*,  *l*_*i*_ ∈ *L*. The matrix *V*^*k*^=(*v*_*ij*_^*k*^)_*n*×*n*_ meets *v*_*ij*_^*k*^ ∈ *L*, *v*_*ij*_^*k*^=*l*_*i*_. There exists a negation operator: *v*_*ji*_^*k*^=neg(*l*_*i*_)=*l*_*g*−*i*_, *v*_*ii*_^*k*^=*l*_*g*/2_
5. *Multiplicative Preference Relations* [69]. Suppose that the evaluator *e*_*k*_ compares every two design alternatives in the set of alternatives *X* under a certain indicator and gives a reciprocal judgment matrix *A*^*k*^=(*a*_*ij*_^*k*^)_*n*×*n*_, where *a*_*ij*_^*k*^ represents the relative importance of the alternative *x*_*i*_ to the alternative *x*_*j*_ that evaluator *e*_*k*_ thinks, and the matrix *A*^*K*^ meets *a*_*ij*_^*k*^ > 0, *a*_*ii*_^*k*^=1, *a*_*ij*_^*k*^ × *a*_*ji*_^*k*^=1
6. *Fuzzy Preference Relations* [70]. Suppose that the evaluator *e*_*k*_ compares every two design alternatives in the set of alternatives *X* under a certain indicator and gives a complementary judgment matrix *P*^*k*^=(*p*_*ij*_^*k*^)_*n*×*n*_, where *p*_*ij*_^*k*^ indicates the degree to which the evaluator *e*_*k*_ thinks that the alternative *x*_*i*_ is better than the alternative *x*_*j*_, and *p*_*ij*_^*k*^ ∈ [0,1], *p*_*ij*_^*k*^+*p*_*ji*_^*k*^=1, *p*_*ii*_^*k*^=0.5

**Step 3 .**

Uniforming different preference representation forms.

With multiple preference forms being in presence, it is necessary to uniform different preference information so as to effectively aggregate group opinions and select the optimal design alternative. In this study, we consider uniforming the above-mentioned preference structures into a complementary judgment matrix.

We use the following function [71] to transform the utility value of *x*_*i*_ into a complementary judgment matrix.

(3) $$\begin{array}{c} p_{ij}^{k}=f_{2}\left(u_{i}^{k},u_{j}^{k}\right)=\frac{{\left(u_{i}^{k}\right)}^{2}}{{\left(u_{i}^{k}\right)}^{2}+{\left(u_{j}^{k}\right)}^{2}}. \end{array}$$

We use the following function [72] to transform the preference order of *x*_*i*_ into a complementary judgment matrix.

(4) $$\begin{array}{c} p_{ij}^{k}=f_{1}\left(o_{i}^{k},o_{j}^{k}\right)=\frac{1}{2}\left[1+\frac{o_{j}^{k}-o_{i}^{k}}{n-1}\right]. \end{array}$$

We use the following function [73] to transform the evaluation value of the interval number of *x*_*i*_ into a complementary judgment matrix.

(5) $$\begin{array}{c} p_{ij}^{k}=f_{4}\left(d_{ij}^{k}\right)=\max\left\{1-\max\left(\frac{d_{j}^{u^{k}}-d_{i}^{l^{k}}}{d_{i}^{u^{k}}-d_{i}^{l^{k}}+d_{j}^{u^{k}}-d_{j}^{l^{k}}},0\right),0\right\}. \end{array}$$

We use the following function [74] to transform the linguistic evaluation matrix of *x*_*i*_ into a complementary judgment matrix.

(6) $$\begin{array}{c} p_{ij}^{k}=f_{5}\left(v_{ij}^{k}\right)=\frac{{\left(g/2\right)}^{\left(I\left(v_{ij}^{k}\right)/g/2\right)}}{{\left(g/2\right)}^{\left(I\left(v_{ij}^{k}\right)/g/2\right)}+{\left(g/2\right)}^{\left(I\left(v_{ij}^{k}\right)/g/2\right)}}. \end{array}$$

We use the following function [71] to transform the reciprocal judgment matrix of *x*_*i*_ into a complementary judgment matrix.

(7) $$\begin{array}{c} p_{ij}^{k}=f_{3}\left(a_{ij}^{k}\right)=\frac{1}{2}\left(1+\;\log_{9}a_{ij}^{k}\right). \end{array}$$

**Step 4 .**

Preference aggregation.

After all preference structures are uniformed into complementary judgment matrices, we aggregate the preference information of each evaluator into group preference information. This paper uses the HWA operator [75] for processing, which not only considers the importance of the position of each data but also reflects the importance of the data itself.

We use the HWA operator to aggregate the vector (*p*_*ij*_^1^, *p*_*ij*_^2^,…, *p*_*ij*_^*k*^) and obtain the comprehensive evaluation value *P*_*ij*_^*c*_*j*_^ of *k* evaluators on indicator *i*_*j*_ of design alternative *x*_*i*_:

(8) $$\begin{array}{c} P_{ij}^{c_{j}}={\text{HWA}}_{w,\omega }\left(p_{ij}^{1},p_{ij}^{2},\ldots ,p_{ij}^{k}\right)=\sum_{s=1}^{k}\omega_{s}v_{s}, \end{array}$$

where *v*_*s*_ is the element that ranks *s* in a decreasing order in a set of weighted data (*λw*_1_*x*_*ij*_1__^1^, *λw*_2_*x*_*ij*_1__^2^,…, *λw*_*l*_*x*_*ij*_1__^*l*^), where *W*=(*w*_1_, *w*_2_,…,*w*_*l*_)^*T*^ is the evaluator weight vector, and*ω*=(*ω*_1_, *ω*_2_,…,*ω*_*k*_)^*T*^ is determined by the fuzzy linguistic quantifier. *λ* is the balance factor.

(9) $$\begin{array}{c} w_{i}=\theta \left(\frac{i}{n}\right)-\theta \left(\frac{\left(i-1\right)}{n}\right),\;i=1,2,\ldots ,n. \end{array}$$

The fuzzy linguistic quantifier *θ* is given by the following equation [76]:

(10) $$\begin{array}{c} \theta \left(r\right)=\left\{\begin{array}{cc} 0, & r<a,\\ \frac{r-a}{b-a}, & a\leq r\leq b,\\ 1, & r>b, \end{array}\right) \end{array}$$

where *a*, *b*, *r* ∈ [0,1], under the principles of “at least half,” “most,” and “as many as possible,” the corresponding parameters (*a*, *b*) to the fuzzy linguistic quantifier *Q*(*r*) are (0, 0.5), (0.3, 0.8), and (0.5, 1) [77, 78].

**Step 5 .**

Consensus measurement.

The calculation of the consensus degree is based on the comparison between each individual evaluator's ranking of the alternatives and the evaluator group's ranking of the alternatives. By comparing the rankings of a given alternative, the approximate degree *p*_*k*_(*x*_*i*_) of the number *x*_*i*_ alternative of the individual evaluator *e*_*k*_ is calculated.

(11) $$\begin{array}{c} p_{k}\left(x_{i}\right)={\left(\alpha \cdot \left|V_{i}^{c_{j}}-V_{i}^{k}\right|\right)}^{b}\in \left[0,1\right], \end{array}$$

where *V*_*k*_=(*V*_1_^*k*^, *V*_2_^*k*^,…, *V*_*n*_^*k*^) is the ranking provided by the evaluator *e*_*k*_, *V*_*i*_^*k*^ is the ranking provided by the individual evaluator *e*_*k*_ on the alternative *x*_*i*_ under the indicator *i*_*j*_, *V*_*c*_*j*__=(*V*_1_^*c*_*j*_^, *V*_2_^*c*_*j*_^,…, *V*_*n*_^*c*_*j*_^) is the ranking provided by the evaluator group, and *V*_*i*_^*c*_*j*_^ is the ranking provided by the evaluator group on the alternative *x*_*i*_ under the indicator *i*_*j*_. Particularly, we take *α*=1/(*n* − 1). The parameter *b* controls the rigor of the consensus process. The closer the value of *b* is to 1, the lower the rigor is, and the fewer rounds of discussion among the evaluator group are needed. The closer the value of *b* is to 0, the higher the rigor is, and the more rounds of discussion among the evaluator group are needed.

1 − *p*_*k*_(*x*_*i*_) is the approximate value between the evaluator *e*_*k*_'s evaluation of alternative *x*_*i*_ and the group's evaluation. We calculate the consensus of all evaluators on the number *x*_*i*_ alternative:

(12) $$\begin{array}{c} C\left(x_{i}\right)=1-\sum_{k=1}^{l}\frac{p_{k}\left(x_{i}\right)}{l}. \end{array}$$

We calculate the consensus degree *C*_*X*_ among all evaluators on the alternative set *X*. When there is only one optimal solution *x*_*s*_ in the solution set [79], we obtain the value of *C*_*X*_ by aggregating *C*(*x*_*i*_):

(13) $$\begin{array}{c} C_{X}=\left(1-\beta \right)\frac{1}{n}\sum_{i=1}^{n}C\left(x_{i}\right)+\beta C\left(x_{s}\right). \end{array}$$

**Step 6 .**

Adjust preferences based on the feedback information.

When the consensus degree *C*_*X*_ does not reach the predetermined level, the evaluators are asked to revise their preferences. In this case, there are three issues to be considered: (1) which evaluators need to modify their opinions, (2) which elements need to be modified, and (3) what is the direction of the modification.

We identify the evaluators that need to modify some of their preference information by calculating the approximate degree. The approximate degree *P*_*x,k*_ of the evaluator *e*_*k*_ is obtained through the aggregation of the approximation of each design alternative.

(14) $$\begin{array}{c} P_{x,k}=\left(1-\beta \right)\frac{1}{n}\sum_{i=1}^{n}\left(1-p_{k}\left(x_{i}\right)\right)+\beta \left(1-p_{k}\left(x_{s}\right)\right). \end{array}$$

The evaluators are ranked based on the approximation value *P*_*x,k*_, *k* = 1, 2,…,*l*. Every evaluator knows his/her position and approximation of each alternative. The evaluator with the smaller value of *P*_*x,k*_ or the evaluator whose rank is at the lower end needs to modify their preference. Now, we set a threshold *p*, *p*∈[0, 1] to determine how many evaluators we need to modify their preference. If *P*_*x,k*_ ＜ *p*, then *e*_*k*_ needs to modify his/her preference opinion. In this study, we set *P*=0.75 [80].

For evaluators who need to modify their preference, if *V*_*i*_^*c*_*j*_^ > *V*_*i*_^*k*^, then *e*_*k*_ needs to improve his/her appraisal of alternative *x*_*i*_; if *V*_*i*_^*c*_*j*_^ < *V*_*i*_^*k*^, *e*_*k*_ needs to lower his/her appraisal for alternative *x*_*i*_; if *V*_*i*_^*c*_*j*_^=*V*_*i*_^*k*^, *e*_*k*_'s evaluation of alternative *x*_*i*_ remains unchanged.

**Step 7 .**

Calculate the ranking of the alternatives.

We use the HWA operator to aggregate the vectors (*p*_*ij*_^*c*_1_^, *p*_*ij*_^*c*_2_^,…, *p*_*ij*_^*c*_*m*_^) and obtain the comprehensive evaluation value of the alternative *x*_*i*_ by *k* evaluators which is *P*_*ij*_^*C*^ under *m* indicators:

(15) $$\begin{array}{c} P_{ij}^{C}={\text{HWA}}_{z,\omega }\left(p_{ij}^{c_{1}},p_{ij}^{c_{2}},\ldots ,p_{ij}^{c_{m}}\right)=\sum_{s=1}^{m}\omega_{s}v_{s}, \end{array}$$

where *v*_*s*_ is the number *s* largest element in the set of weighted data (*λz*_1_*x*_*ij*_^1^, *λz*_2_*x*_*ij*_^2^,…, *λz*_*m*_*x*_*ij*_^*m*^), where *Z*=(*z*_1_, *z*_2_,…,*z*_*m*_)^*T*^ is the indicator weight vector, and *ω*=(*ω*_1_, *ω*_2_,…,*ω*_*k*_)^*T*^ is determined by the fuzzy linguistic quantifier.

## 4. A Numerical Example

In this section, the proposed model is applied in the evaluation of MFIAs. At first, the background of the evaluation of MFIAs is proposed. Then, the proposed method is used to evaluate the alternatives. Finally, the feasibility and rationality of the proposed method are demonstrated by sensitivity analysis and comparative analysis.

### 4.1. Background

Fluorescence immunochromatography is usually used for high sensitivity detection or rapid detection, which is promising and practical. And it is used to detect human serum, plasma, whole blood, and urine samples. The results are mostly applied as the biological basis of diagnosis for myocardial injury, heart failure, cardiovascular inflammation, acute and chronic nephropathy, and other diseases. Immunochromatography Assay is an analytical method combining immunoassay methods and chromatographic methods, with characteristics of strong peculiarity, simple operation, and rapid detecting.

At present, most of the fluorescence immune tomography analyzers are single-channel equipment, and it is difficult to realize the simultaneous detection of a single sample with multiple items. The efficiency is low. When the sample size is large, it is difficult to meet the detection requirements. For this reason, five MFIAs are designed. The whole mechanical part is composed of a reagent card placement structure, scanning detection structure, and guide rail structure. Six channels are designed, and a single-layer structure mode is adopted. The evaluation of the five MFIAs is conducted to verify the effectiveness of the methods proposed.

### 4.2. The Evaluation of MFIAs

**Figure 2:**
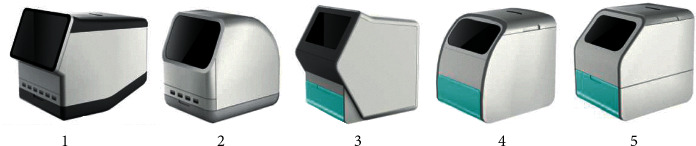
Design alternatives.

We exemplify the approach through the evaluation of the design alternatives of MFIAs and elaborate the implementation results of the proposed approach as follows. After conducting the preliminary investigation, analysis, and design, 3 product designers proposed a total of 5 alternatives for evaluation (see Figure 2).

An evaluator group *E* = {*e*_1_, *e*_2_, *e*_3_, *e*_4_, *e*_5_, *e*_6_, *e*_7_, *e*_8_} with 2 senior product designers, 2 engineers, 1 project leader, 1 customer representative, and 2 product users participated in this project. After researching users' demands, integrating the results of group discussions, we finalized the indicators of the MFIAs (see Table 1).

**Step 8 .**

Use the AHP method to calculate the weights of evaluators and the weights of the indicators and the result is listed as follows: the weight vector of 8 evaluators is *W* = {0.17, 0.17, 0.18, 0.16, 0.06, 0.12, 0.07, 0.07}, and the weight vector of the 6 evaluation indicators is *Z* = {0.08, 0.10, 0.19, 0.15, 0.25, 0.23}. The consensus threshold is set to be 0.75.

**Step 9 .**

For indicator *i*_1_, 8 evaluators evaluate the 5 alternatives in the following way: *e*_1_ and *e*_2_ use reciprocal judgment matrix, *e*_3_ and *e*_4_ use complementary judgment matrix, *e*_5_ uses utility value, *e*_6_ uses interval number evaluation value, *e*_7_ uses linguistic evaluation value, and *e*_8_ uses preference ordering. The initial evaluation results collected are as follows:

(16) $$\begin{array}{c} A^{1}=\left(\begin{array}{ccccc} 1 & \frac{1}{3} & \frac{1}{5} & 1\frac{1}{3} & \frac{1}{5}\\ 3 & 1 & \frac{1}{3} & 1 & \frac{1}{3}\\ 5 & 3 & 1 & 3 & 1\\ 3 & 1 & \frac{1}{3} & 1 & \frac{1}{3}\\ 5 & 3 & 1 & 3 & 1 \end{array}\right),\\ A^{2}=\left(\begin{array}{ccccc} 1 & \frac{1}{5} & 1 & \frac{1}{5} & \frac{1}{5}\\ 5 & 1 & 3 & 3 & \frac{1}{3}\\ 1 & \frac{1}{3} & 1 & 3 & \frac{1}{3}\\ 5 & \frac{1}{3} & \frac{1}{3} & 1 & \frac{1}{3}\\ 5 & 3 & 3 & 3 & 1 \end{array}\right),\\ P^{3}=\left(\begin{array}{ccccc} 0.5 & 0.3 & 0.5 & 0.1 & 0.1\\ 0.7 & 0.5 & 0.7 & 0.3 & 0.3\\ 0.5 & 0.3 & 0.5 & 0.3 & 0.3\\ 0.9 & 0.7 & 0.7 & 0.5 & 0.3\\ 0.9 & 0.7 & 0.7 & 0.7 & 0.5 \end{array}\right),\\ P^{4}=\left(\begin{array}{ccccc} 0.5 & 0.3 & 0.3 & 0.1 & 0.1\\ 0.7 & 0.5 & 0.7 & 0.3 & 0.3\\ 0.7 & 0.3 & 0.5 & 0.3 & 0.3\\ 0.9 & 0.7 & 0.7 & 0.5 & 0.5\\ 0.9 & 0.7 & 0.7 & 0.5 & 0.5 \end{array}\right),\\ U^{5}=\left\{0.6,0.7,0.8,0.5,0.4\right\},\\ D^{6}=\left\{\left[0.6,0.8\right],\left[0.4,0.6\right],\left[0.7,0.9\right],\left[0.8,0.9\right],\left[0.8,0.9\right]\right\},\\ V^{7}=\left(\begin{array}{ccccc} l_{2} & l_{1} & l_{2} & l_{1} & l_{1}\\ l_{3} & l_{2} & l_{1} & l_{1} & l_{1}\\ l_{2} & l_{3} & l_{2} & l_{3} & l_{2}\\ l_{3} & l_{3} & l_{1} & l_{2} & l_{1}\\ l_{3} & l_{3} & l_{2} & l_{3} & l_{2} \end{array}\right),\\ O^{8}=\left\{2,5,1,3,4\right\}. \end{array}$$

**Step 10 .**

According to equations (3)–(7), all different preference structures are transformed into complementary judgment matrices:

(17) $$\begin{array}{c} P_{1}=\left(\begin{array}{ccccc} 0.500 & 0.250 & 0.134 & 0.250 & 0.134\\ 0.750 & 0.500 & 0.250 & 0.500 & 0.250\\ 0.866 & 0.750 & 0.500 & 0.750 & 0.500\\ 0.750 & 0.500 & 0.250 & 0.500 & 0.250\\ 0.866 & 0.750 & 0.500 & 0.750 & 0.500 \end{array}\right),\\ P_{2}=\left(\begin{array}{ccccc} 0.500 & 0.134 & 0.500 & 0.134 & 0.134\\ 0.866 & 0.500 & 0.750 & 0.750 & 0.250\\ 0.500 & 0.250 & 0.500 & 0.750 & 0.250\\ 0.866 & 0.250 & 0.250 & 0.500 & 0.250\\ 0.866 & 0.750 & 0.750 & 0.750 & 0.500 \end{array}\right),\\ P_{5}=\left(\begin{array}{ccccc} 0.500 & 0.424 & 0.360 & 0.590 & 0.692\\ 0.576 & 0.500 & 0.434 & 0.662 & 0.754\\ 0.640 & 0.566 & 0.500 & 0.719 & 0.800\\ 0.410 & 0.338 & 0.281 & 0.500 & 0.610\\ 0.308 & 0.246 & 0.200 & 0.390 & 0.500 \end{array}\right),\\ P_{6}=\left(\begin{array}{ccccc} 0.500 & 1.000 & 0.250 & 0.000 & 0.000\\ 0.000 & 0.500 & 0.000 & 0.000 & 0.000\\ 0.750 & 1.000 & 0.500 & 0.333 & 0.333\\ 1.000 & 1.000 & 0.667 & 0.500 & 0.500\\ 1.000 & 1.000 & 0.667 & 0.500 & 0.500 \end{array}\right),\\ P_{7}=\left(\begin{array}{ccccc} 0.500 & 0.333 & 0.500 & 0.333 & 0.333\\ 0.667 & 0.500 & 0.333 & 0.333 & 0.333\\ 0.500 & 0.667 & 0.500 & 0.500 & 0.500\\ 0.667 & 0.667 & 0.500 & 0.500 & 0.333\\ 0.667 & 0.667 & 0.500 & 0.500 & 0.500 \end{array}\right),\\ P_{8}=\left(\begin{array}{ccccc} 0.500 & 0.875 & 0.375 & 0.625 & 0.750\\ 0.125 & 0.500 & 0.000 & 0.250 & 0.375\\ 0.625 & 1.000 & 0.500 & 0.750 & 0.875\\ 0.375 & 0.750 & 0.250 & 0.500 & 0.625\\ 0.250 & 0.625 & 0.125 & 0.375 & 0.500 \end{array}\right). \end{array}$$

**Step 11 .**

According to equations (9) and (10), we use the HWA operator and the fuzzy majority criterion based on the fuzzy quantization operator “as many as possible.” *ω*=(0,0,0,0,0.25, 0.25, 0.25, 0.25)^*T*^; the weight of the evaluator is *W*=(0.17, 0.18, 0.18, 0.15, 0.06, 0.12, 0.07, 0.07)^*T*^. According to equation (8), the group evaluation value obtained by using the HWA operator is

(18) $$\begin{array}{c} P^{c_{1}}=\left(\begin{array}{ccccc} 0.320 & 0.228 & 0.201 & 0.105 & 0.113\\ 0.180 & 0.320 & 0.099 & 0.161 & 0.184\\ 0.404 & 0.342 & 0.320 & 0.332 & 0.331\\ 0.435 & 0.324 & 0.224 & 0.320 & 0.290\\ 0.405 & 0.434 & 0.272 & 0.289 & 0.320 \end{array}\right). \end{array}$$

**Step 12 .**

Use the fuzzy majority criterion based on the fuzzy quantization operator “as many as possible” to calculate the ranking of the complementary judgment matrix, where *ω*=(0,0,0.2, 0.4, 0.4)^*T*^, and the evaluators' ranking of the 5 alternatives on indicator 1 is as follows:

(19) $$\begin{array}{c} e_{1}:x_{3}=x_{5}>x_{2}=x_{4}>x_{1},\\ e_{2}:x_{5}>x_{2}>x_{3}>x_{4}>x_{1},\\ e_{3}:x_{5}>x_{4}>x_{2}>x_{3}>x_{1},\\ e_{4}:x_{4}=x_{5}>x_{2}>x_{3}>x_{1},\\ e_{5}:x_{3}>x_{2}>x_{1}>x_{4}>x_{5},\\ e_{6}:x_{4}=x_{5}>x_{3}>x_{1}>x_{2},\\ e_{7}:x_{3}=x_{5}>x_{4}>x_{2}>x_{1},\\ e_{8}:x_{3}>x_{1}>x_{4}>x_{5}>x_{2}. \end{array}$$

Similarly, for indicator *i*_1_, the evaluator group thinks that the ranking of the 5 alternatives is *x*_3_ > *x*_5_ > *x*_4_ > *x*_2_ > *x*_1_. This ranking shows that the third alternative is the best under indicator *i*_1_.

Use equation (11) to calculate the individual evaluator's degree of approximation *p*_*k*_(*x*_*i*_) for each design alternative. First, we calculate *V*_*i*_^*c*_*j*_^ − *V*_*i*_^*k*^ (see Table 2). For example, for the second element in the first row, the evaluator *e*_1_ ranks *x*_2_ and *x*_4_ in the 3rd place, while the group ranks *x*_2_ in the 4th place and *x*_4_ in the 3rd place; then, we have *V*_2_^*c*_1_^ − *V*_2_^1^=−1 and *V*_4_^*c*_1_^ − *V*_4_^1^=0. When taking *a*=1/(*n* − 1), *b*=0.7, *p*_*k*_(*x*_*i*_) is calculated according to equation (11) and the results are obtained (see Table 3).

Then, we calculate the degree of consensus of all evaluators on the set of alternatives according to equation (13):*C*_*X*_=(1 − *β*)((0.773+0.609+0.642+0.704+0.537)/5)+*β* × 0.642

When taking *β*=0.8, the consensus degree is *C*_*X*_=0.644 < *γ*=0.75, and some evaluators need to modify their preference opinions.

**Step 13 .**

Evaluators modify their preferences according to the feedback mechanism.

By calculating the degree of approximation using equation (14), evaluators who need to modify some of their preferences are identified.

(20) $$\begin{array}{c} P_{1,1}=0.848+0.152\beta ,\\ P_{1,2}=0.602-0.218\beta ,\\ P_{1,3}=0.609-0.427\beta ,\\ P_{1,4}=0.562-0.379\beta ,\\ P_{1,5}=0.514+0.486\beta ,\\ P_{1,6}=0.526-0.142\beta ,\\ P_{1,7}=0.924+0.076\beta ,\\ P_{1,8}=0.638+0.362\beta . \end{array}$$

Suppose *β*=0.8; the degree of approximation of the evaluators is sorted from high to low: *e*_7_, *e*_1_, *e*_8_, *e*_5_, *e*_2_, *e*_6_, *e*_3_, and *e*_4_.

In this paper, we suppose *p*=0.75. Then, the two evaluators *e*_3_ and *e*_*4*_ with a low degree of approximation need to modify their evaluation. According to the proposed rules, their reevaluation results are

(21) $$\begin{array}{c} P_{3}=\left(\begin{array}{ccccc} 0.5 & 0.3 & 0.3 & 0.1 & 0.1\\ 0.7 & 0.5 & 0.3 & 0.3 & 0.3\\ 0.7 & 0.7 & 0.5 & 0.5 & 0.5\\ 0.9 & 0.7 & 0.5 & 0.5 & 0.3\\ 0.9 & 0.7 & 0.5 & 0.7 & 0.5 \end{array}\right),\\ p_{4}=\left(\begin{array}{ccccc} 0.5 & 0.3 & 0.3 & 0.1 & 0.1\\ 0.7 & 0.5 & 0.3 & 0.3 & 0.3\\ 0.7 & 0.7 & 0.5 & 0.7 & 0.5\\ 0.9 & 0.7 & 0.3 & 0.5 & 0.5\\ 0.9 & 0.7 & 0.5 & 0.5 & 0.5 \end{array}\right). \end{array}$$

After obtaining the reevaluation results, we perform the second round of calculations. Similar to the calculation process of the first round, we obtain the consensus degree (0.787) from the second round, which now meets the requirements. Therefore, we take the evaluation results of the second round as the final decision-making basis.

(22) $$\begin{array}{c} P^{c_{1}}=\left(\begin{array}{ccccc} 0.320 & 0.228 & 0.201 & 0.105 & 0.113\\ 0.180 & 0.320 & 0.099 & 0.161 & 0.184\\ 0.404 & 0.386 & 0.320 & 0.341 & 0.331\\ 0.435 & 0.325 & 0.224 & 0.320 & 0.290\\ 0.405 & 0.434 & 0.272 & 0.289 & 0.320 \end{array}\right). \end{array}$$

In the same way, we proceed to Steps 2–6 to obtain the evaluation results of the other 5 indicators.

(23) $$\begin{array}{c} P^{c_{2}}=\left(\begin{array}{ccccc} 0.320 & 0.147 & 0.073 & 0.084 & 0.095\\ 0.477 & 0.320 & 0.225 & 0.269 & 0.262\\ 0.450 & 0.315 & 0.320 & 0.220 & 0.246\\ 0.492 & 0.267 & 0.259 & 0.320 & 0.269\\ 0.485 & 0.290 & 0.325 & 0.311 & 0.320 \end{array}\right),\\ P^{c_{3}}=\left(\begin{array}{ccccc} 0.320 & 0.211 & 0.188 & 0.123 & 0.203\\ 0.253 & 0.320 & 0.150 & 0.125 & 0.155\\ 0.345 & 0.318 & 0.320 & 0.268 & 0.254\\ 0.398 & 0.443 & 0.235 & 0.320 & 0.331\\ 0.375 & 0.399 & 0.168 & 0.223 & 0.320 \end{array}\right),\\ P^{c_{4}}=\left(\begin{array}{ccccc} 0.320 & 0.065 & 0.136 & 0.016 & 0.068\\ 0.472 & 0.320 & 0.261 & 0.159 & 0.164\\ 0.372 & 0.275 & 0.320 & 0.099 & 0.132\\ 0.403 & 0.284 & 0.260 & 0.320 & 0.369\\ 0.361 & 0.232 & 0.201 & 0.260 & 0.320 \end{array}\right),\\ P^{c_{5}}=\left(\begin{array}{ccccc} 0.320 & 0.339 & 0.219 & 0.259 & 0.180\\ 0.146 & 0.320 & 0.080 & 0.216 & 0.091\\ 0.386 & 0.405 & 0.320 & 0.413 & 0.295\\ 0.214 & 0.329 & 0.148 & 0.320 & 0.233\\ 0.316 & 0.353 & 0.250 & 0.327 & 0.320 \end{array}\right),\\ P^{c_{6}}=\left(\begin{array}{ccccc} 0.320 & 0.495 & 0.271 & 0.298 & 0.353\\ 0.129 & 0.320 & 0.104 & 0.245 & 0.207\\ 0.334 & 0.402 & 0.320 & 0.363 & 0.378\\ 0.148 & 0.355 & 0.122 & 0.320 & 0.252\\ 0.178 & 0.418 & 0.167 & 0.291 & 0.320 \end{array}\right). \end{array}$$

**Step 14 .**

We use the HWA operator and equation (15) to calculate, in which the indicator weight is *Z*=(0.08, 0.10, 0.19, 0.15, 0.25, 0.23)^*T*^. By leveraging the fuzzy majority criterion based on the principle of fuzzy quantization operator, i.e., considering as many indicators as possible, *ω*=(0,0,0, (1/3), (1/3), (1/3))^*T*^, it is ensured that a design alternative performs well under the indicators as many as possible. Then, we get the overall evaluation and ranking of the 5 alternatives under all 6 indicators.

(24) $$\begin{array}{c} P=\left(\begin{array}{ccccc} 0.211 & 0.085 & 0.088 & 0.038 & 0.058\\ 0.161 & 0.211 & 0.104 & 0.121 & 0.124\\ 0.266 & 0.207 & 0.211 & 0.128 & 0.142\\ 0.236 & 0.190 & 0.144 & 0.211 & 0.211\\ 0.244 & 0.197 & 0.168 & 0.186 & 0.211 \end{array}\right). \end{array}$$

The alternatives are arranged in descending order: *x*_5_≻*x*_4_≻*x*_3_≻*x*_2_≻*x*_1_.

### 4.3. Sensitivity Analysis

Considering that the ranking of alternatives can be affected by the indicator weights, the sensitivity of the proposed method is analyzed and discussed in this section. Specifically, we increase each indicator by 30% and 60%, respectively, and then reduce it by 30% and 60%, respectively. When a particular indicator increases or decreases, other indicators also change proportionally, so that the sum of indicator weights is equal to 1. Twenty-four experiments are conducted, and the rankings of the 24 experiments are demonstrated (see Table 4).

**Figure 3:**
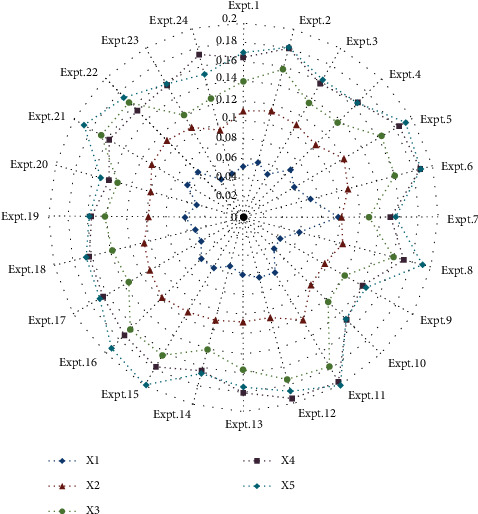
Result of sensitivity analysis.

According to the results of sensitivity analysis (see Figure 3), it is easy to find that, among the 18 experiments, *x*_5_ is better than the other four alternatives accounting for 75% of the total number of experiments, which shows that *x*_5_ is the best alternative of MFIAs. In 22 experiments, *x*_4_ is better than *x*_3_, accounting for 91.7% of the total experiments. *x*_2_ has been ranked fourth in all experiments. *x*_1_ ranks the lowest in all experiments, indicating that it is the worst option for MFIAs. Therefore, we can conclude that *x*_5_ can be recommended as the best alternative from a comprehensive point of view.

Sensitivity analysis indicated that due to different indicator weights, the evaluation of MFIAs produced different results. Therefore, the indicator and weight determination should be more prudent when carrying out the evaluation research of MFIAs to ensure relatively fair and objective evaluation results.

### 4.4. Comparative Analysis

First, through literature review, it can be found that previous evaluation and selection of product design alternatives basically adopt a single preference structure [35, 37, 39], which cannot meet the different needs of evaluators. Apparently, these studies did not take the following factors into account, i.e., the difference in expression habits, educational backgrounds, knowledge, and experiences of different decision-makers. Evaluators usually adopt heterogeneous preference representation structures to express their preference for alternatives. Compared with previous research, this paper studies the evaluation of product design alternatives with multiple preference structures. Evaluators can use multiple preference structures to evaluate product design alternatives. It not only saves the time required for evaluators to adapt to unfamiliar preference structures but also allows evaluators with different expression habits and backgrounds to express their preferences in a more flexible way.

Secondly, TOPSIS [36, 39] and VIKOR [37] are often used to obtain the ranking results of the final alternatives in some existing researches on the evaluation of product design schemes or use the comprehensive coefficient method to get a total score of each scheme and then rank the schemes [35]. However, this method only focuses on the aggregation of individual opinions into group opinions, while ignoring the consensus degree among evaluators. Due to the influence of objective factors such as the uncertainty of things and subjective factors such as the knowledge structure and judgment level of evaluators, the opinions of evaluators often differ greatly. Therefore, it is necessary to consider the level of consensus among decision-makers. In view of this situation, based on the HWA operator and consensus measure, the consensus process is taken into account in the product design evaluation process. It provides effective support for the evaluation of product design alternatives, improves the credibility of the evaluation results, and avoids the consequences of the traditional forcible use of simple aggregate methods to achieve compromise results that are difficult to reflect the true preferences of evaluators.

Finally, multiple preference structures and consensus models were introduced into the evaluation process at the same time. Based on the advantages of multiple preference structures to meet the different needs and expression habits of evaluators, and the advantages of consensus models that can reduce differences of opinion, a comprehensive method combining multiple preference structures and consensus models is proposed. This method provides an effective method for the expression of individual evaluation in the evaluation of product design alternatives and reduces the influence of evaluation group divergence on the evaluation results. It is conducive to the smooth implementation of product design program evaluation and is conducive to building a more harmonious interpersonal relationship within the organization.

## 5. Conclusions

To solve the problem of the inability to express a certain form of information caused by the heterogeneity of the evaluators in the product design evaluation process, avoid the lack of information caused by using the same form of preference, ensure that all evaluators can naturally and accurately express their preferences, and ensure that a certain degree of consensus on the pros and cons of the program is reached in the case of heterogeneity among evaluators, this paper proposes an approach for reaching consensus with mixed preference information on the evaluation of product design alternatives. First, a random evaluator expresses his/her preference for each product design alternative by randomly using a preference representation from these: preference order, utility value, reciprocal judgment matrix, complementary judgment matrix, interval number evaluation value, and linguistic evaluation value, so as to retain the integrity and accuracy of his/her evaluation information. Second, we use the corresponding transfer function to uniform the different preference forms of the evaluators into the form of a complementary judgment matrix. Third, we use the aggregation operator to aggregate the opinions of the evaluators into group opinions and consider the weights of individual evaluators and evaluation indicators. Then, by comparing individual preferences and group preferences, we measure the group consensus and identify outlier opinions. Finally, we ask evaluators with the smaller value or the evaluator whose rank is at the lower end to modify their opinion according to the feedback mechanism until the group reaches a consensus. We then use a product design evaluation case to illustrate the feasibility and effectiveness of the proposed method. The results show that the approach is easy to operate, taking into account the heterogeneity needs among product design evaluators, and rationally uses the preference information provided by the evaluators. In this way, the evaluation results we obtain from the process are more accurate and robust. The approach has strong practical value and provides an important basis for the effective development of design activities.

This research has three main contributions. Firstly, we proposed an approach for reaching a consensus on product design alternative evaluation with mixed preference information reflected. In order to solve the problem of the evaluator group having obvious heterogeneity during the evaluation process, we have allowed for various preference representation forms. Research shows that this method can solve the problem of a single preference structure being unable to meet the requirements of heterogeneous evaluator groups. This approach also makes the evaluation results more reasonable and credible. Secondly, we introduced the HWA operator into the process of aggregating the evaluation results. The HWA operator considers both the importance of the ranking position of each alternative and the importance of the alternative itself, making the aggregation result more accurate. Furthermore, we take into consideration the consensus-reaching process in the evaluation of product design alternatives. This approach provides effective support for the multiobjective evaluation of product design alternatives, improves the credibility of the evaluation results, and avoids the situation where evaluation results are compromised and decision-makers' true preferences are not faithfully reflected when using traditional aggregation methods. At the same time, we make adjustments to the evaluation of the alternatives based on the feedback information, and only by modifying certain individual evaluators' preference opinion, individual decision-makers' heterogeneous characteristics as well as their influence within the group are reflected.

However, the approach proposed in this paper still possesses limitations. In the evaluation process of product design alternatives in real life, the six reference forms mentioned in this paper cannot comprehensively summarize all the forms preferred by evaluators with obvious heterogeneity. Meanwhile, complex linguistic expressions will be considered in future studies due to their increasingly widespread application, such as hesitant fuzzy linguistic information [81], distributed linguistic information [82], HFLTS probability distribution [83], and comparative linguistic expressions [84, 85] and other complex language expressions and more preference forms. Lastly, only 8 decision-makers participated in the research, which is another limitation of this paper. It is our hope that we can solve these problems in future research, focusing on meeting the heterogeneous needs of evaluators in the process of product design evaluation, and obtaining robust product evaluation results.

## Figures and Tables

**Table 1:** Evaluation indicators of the design alternatives.

First-Level indicators	Second-level indicators	Detailed description
*Meeting technical requirements*	*i*_*1*_ easy to assemble and maintain	The assembling method makes it easy to replace the printing paper and it is easy to repair when a failure occurs.
	*i*_*2*_ easy to be processed using plastics	Use plastics processing due to cost constraints.

*Aesthetics of design*	*i*_*3*_ unification between shape and function	The design of functional components is consistent with the overall style.
	*i*_*4*_ conforms to the rule of formal beauty	Well balanced in shape and proportions.

Human-machine interaction coordination	*i*_*5*_ reasonable layout of functions	The layout of these functions is reasonable: a display screen, a port for test paper delivery and recycling, a port for test result printing, switch buttons, etc.
	*i*_*6*_ easy to operate	The angle of the display makes viewing easy; the test paper is easy to deliver and recycle, etc.

**Table 2:** *V*_*i*_^*c*_1_^ − *V*_*i*_^*k*^ calculation results of the 5 alternatives.

*a*	*V*_1_^*c*_1_^ − *V*_1_^*k*^	*V*_2_^*c*_1_^ − *V*_2_^*k*^	*V*_3_^*c*_1_^ − *V*_3_^*k*^	*V*_4_^*c*_1_^ − *V*_4_^*k*^	*V*_5_^*c*_1_^ − *V*_5_^*k*^
*e*_1_	0	−1	0	0	−1
*e*_2_	0	−2	2	1	−1
*e*_3_	0	−1	3	−1	−1
*e*_4_	0	−1	3	−2	−1
*e*_5_	−2	−2	0	1	3
*e*_6_	−1	1	2	−2	−1
*e*_7_	0	0	0	0	−1
*e*_8_	−3	1	0	0	2

**Table 3:** Approximation of individual evaluators for each design alternative.

	*P*_*k*_(*x*_1_)	*P*_*k*_(*x*_2_)	*P*_*k*_(*x*_3_)	*P*_*k*_(*x*_4_)	*P*_*k*_(*x*_5_)
*e*_1_	0.000	0.379	0.000	0.000	0.379
*e*_2_	0.000	0.616	0.616	0.379	0.379
*e*_3_	0.000	0.379	0.818	0.379	0.379
*e*_4_	0.000	0.379	0.818	0.616	0.379
*e*_5_	0.616	0.616	0.000	0.379	0.818
*e*_6_	0.379	0.379	0.616	0.616	0.379
*e*_7_	0.000	0.000	0.000	0.000	0.379
*e*_8_	0.818	0.379	0.000	0.000	0.616

Then, we calculate the consensus degree of each alternative according to equation (12). *C*(*x*_1_) = 0.773, *C*(*x*_2_) = 0.609, *C*(*x*_3_) = 0.642, *C*(*x*_4_) = 0.704, and *C*(*x*_5_) = 0.537.

**Table 4:** Rankings of 24 experiments.

Experiment No.	Ranking orders	Experiment No.	Ranking orders
1	*x*_5_≻*x*_4_≻*x*_3_≻*x*_2_≻*x*_1_	13	*x*_4_≻*x*_5_≻*x*_3_≻*x*_2_≻*x*_1_
2	*x*_5_≻*x*_4_≻*x*_3_≻*x*_2_≻*x*_1_	14	*x*_5_≻*x*_4_≻*x*_3_≻*x*_2_≻*x*_1_
3	*x*_5_≻*x*_4_≻*x*_3_≻*x*_2_≻*x*_1_	15	*x*_5_≻*x*_4_≻*x*_3_≻*x*_2_≻*x*_1_
4	*x*_4_≻*x*_5_≻*x*_3_≻*x*_2_≻*x*_1_	16	*x*_5_≻*x*_4_≻*x*_3_≻*x*_2_≻*x*_1_
5	*x*_5_≻*x*_4_≻*x*_3_≻*x*_2_≻*x*_1_	17	*x*_5_≻*x*_4_≻*x*_3_≻*x*_2_≻*x*_1_
6	*x*_4_≻*x*_5_≻*x*_3_≻*x*_2_≻*x*_1_	18	*x*_5_≻*x*_4_≻*x*_3_≻*x*_2_≻*x*_1_
7	*x*_5_≻*x*_4_≻*x*_3_≻*x*_2_≻*x*_1_	19	*x*_5_≻*x*_4_≻*x*_3_≻*x*_2_≻*x*_1_
8	*x*_5_≻*x*_4_≻*x*_3_≻*x*_2_≻*x*_1_	20	*x*_5_≻*x*_4_≻*x*_3_≻*x*_2_≻*x*_1_
9	*x*_5_≻*x*_4_≻*x*_3_≻*x*_2_≻*x*_1_	21	*x*_5_≻*x*_3_≻*x*_4_≻*x*_2_≻*x*_1_
10	*x*_4_≻*x*_5_≻*x*_3_≻*x*_2_≻*x*_1_	22	*x*_5_≻*x*_3_≻*x*_4_≻*x*_2_≻*x*_1_
11	*x*_5_≻*x*_4_≻*x*_3_≻*x*_2_≻*x*_1_	23	*x*_5_≻*x*_4_≻*x*_3_≻*x*_2_≻*x*_1_
12	*x*_4_≻*x*_5_≻*x*_3_≻*x*_2_≻*x*_1_	24	*x*_4_≻*x*_5_≻*x*_3_≻*x*_2_≻*x*_1_

## Data Availability

The data used to support the findings of this study are available from the corresponding author upon request.
